# 3D modeling of dual-gate FinFET

**DOI:** 10.1186/1556-276X-7-625

**Published:** 2012-11-13

**Authors:** Samson Mil’shtein, Lalitha Devarakonda, Brian Zanchi, John Palma

**Affiliations:** 1 Advanced Electronic Technology Center, ECE Department, University of Massachusetts, Lowell, MA, 01854, USA

**Keywords:** FinFET, Field tailoring, Dual gate, Uniform transconductance.

## Abstract

The tendency to have better control of the flow of electrons in a channel of field-effect transistors (FETs) did lead to the design of two gates in junction field-effect transistors, field plates in a variety of metal semiconductor field-effect transistors and high electron mobility transistors, and finally a gate wrapping around three sides of a narrow fin-shaped channel in a FinFET. With the enhanced control, performance trends of all FETs are still challenged by carrier mobility dependence on the strengths of the electrical field along the channel. However, in cases when the ratio of FinFET volume to its surface dramatically decreases, one should carefully consider the surface boundary conditions of the device. Moreover, the inherent non-planar nature of a FinFET demands 3D modeling for accurate analysis of the device performance. Using the Silvaco modeling tool with quantization effects, we modeled a physical FinFET described in the work of Hisamoto et al. (IEEE Tran. Elec. Devices 47:12, 2000) in 3D. We compared it with a 2D model of the same device. We demonstrated that 3D modeling produces more accurate results. As 3D modeling results came close to experimental measurements, we made the next step of the study by designing a dual-gate FinFET biased at *V*_g1_ >*V*_g2_. It is shown that the dual-gate FinFET carries higher transconductance than the single-gate device.

## Background

Accuracy of modeling of any semiconductor device is an issue of the production cost at its onset. Due to the high cost of VLSI fabrication, there is no room for inaccurate modeling. The widely used commercial package of semiconductor device design named Silvaco offers 2D and 3D modeling options along with quantization effects. For the planar technology, the 2D modeling is the main design tool. However, for non-planar devices such as fin-shaped field-effect transistors (FinFETs), the question remains open on which 2D or 3D versions are more reliable. In the current study, we examined both modeling tools and found that 3D modeling is more accurate. We selected a very simple way to judge our results by modeling an existing FinFET which was produced not by our group but by researchers at UC Berkley [1]. Their FinFET was tested after fabrication, and we compared our modeling results with the actual performance characteristics of the transistor.

The historic tendency to improve control on electron flow along the field-effect transistors (FETs) carries few important milestones. The self-aligned technology provided a design, where one or more gates were extended from the source to the drain [2-4]. The design of junction field-effect transistor with two gates, controlling the channel of FET from top and bottom sides of the channel, was another improvement of the control needed along the FET [5]. Later on, usage of field plates allowed the reduction of the size of individual plates, while improving gate control from source to drain [6]. Finally, the FinFET configuration offered a gate wrapped around a channel [1,7-10]. Discussion of cylindrical gates around quantum wire transistors is out of the scope of our study.

With the gate surrounding the conducting channel on three sides, 3D modeling is needed in order to better understand the operation of a device. To apply 2D modeling, we used the gates only on vertical sides of the ‘fin’-shaped channel. We demonstrated that 2D modeling, in spite of being sensitive to the fin height, gives results which are contradicting the experimental measurements. The 3D modeling produced output characteristics which are very close to the experimental measurements, with little adjustments in the metal work function and field-dependent mobility model.

In recent years we studied the performance of metal semiconductor field-effect transistors (MESFETs) and high electron mobility transistors (HEMTs) manufactured by semiconductor companies, where the design was based on our novel concept of tailoring the electrical field along a channel of FETs. Our 2D modeling of these planar devices did allow significant improvement of transconductance [4,6,10]. The field tailoring principle discussed in [6,10] was applied to 3D modeling. In this novel FinFET, the shaping of the electrical field was controlled not by one but by two wrapped gates. To summarize the above, the current study targeted to check the accuracy of 2D vs. 3D modeling and to use accurate 3D models for the design of a novel, dual-gate FinFET.

## Methods

### Device structure

**Figure 1:**
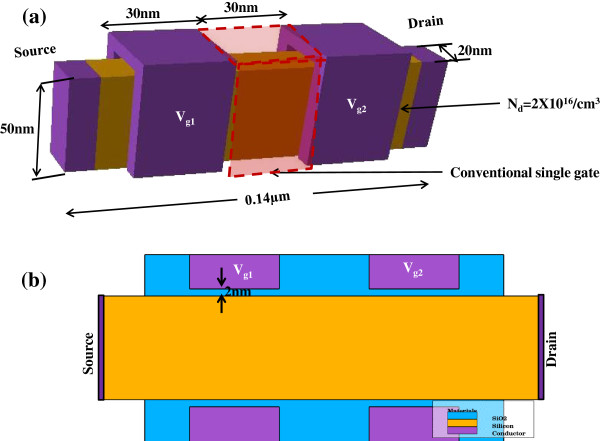
**FinFET structure.** (**a**) 3D view and (**b**) 2D view.

This study uses a single-gate FinFET fabricated by the UC Berkley research group [1]. It had a channel cross section of 50 × 20 nm and length of 0.14 μm. The channel was n-doped to 2 × 10^16^ cm^−3^. The gate length was 30 nm. A 2 nm-thick (20 Å) SiO_2_ layer separated the gate from the Si channel. The wrapped gate surrounded the channel on three sides (Figure 1a), depleting the channel in three directions. The thickness and height of this FinFET were that 2D quantum confinement was possible in the channel. The 3D model gave a result which is similar to the actual FinFET after minor adjustment of the gate work function and field-dependent mobility model.

## Results and discussion

### Comparison of 3D and 2D models

The field distribution inside the channel is the major factor that modifies the direct current (DC) characteristics in any field-effect device. It is expected that the overall field magnitude in the channel is lesser for the 2D model than its 3D equivalent. This is because 2D does not account for the transverse component of the field. We obtained field profiles along the channel for 3D with wrapped gate, with gate on either side of the channel without the top segment and 2D models. Further, FinFET with the wrapped gate and without the top gate segment were modeled in 2D as in Figure 1b.

**Figure 2:**
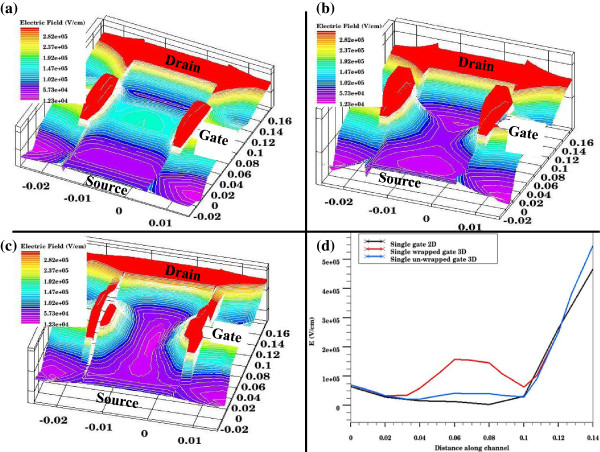
**Electric field profiles.** (**a**) Electric field at the top of the channel for 3D single-gate FinFET with wrapped gate. (**b**) Electric field at the top of the channel for 3D single-gate FinFET with gates on either sides and no top segment. (**c**) Electric field for 2D single-gate FinFET. (**d**) Comparison of field profiles along the middle of the channel.

Comparing electrical field profiles at the top of the channel obtained from 3D models for FinFET with (Figure 2a) and without the top segment (Figure 2b) of the gate, we observed the following:

1. Wrapped gate creates a stronger field at the top than the field at the top of the FinFET without the horizontal gate segment.

2. At the bottom of the channel, both models have similar electrical field distribution.

Comparing in general the electrical field profiles obtained from 3D models (Figure 2a,b) with that of the 2D model (Figure 2c), we observed the following:

1. The electric field strength of 3D models is higher than that of the 2D model as expected.

2. The 3D model with wrapped gate shows the effect for top gate segment which cannot be modeled in a 2D profile.

Figure 2d shows the comparison of strength of the electrical field along a straight line in the middle of the channel. It demonstrates that the average electrical field is stronger for 3D FinFET with wrapped gate. In summary, 3D modeling seems to produce a stronger electrical field than 2D as expected.

**Figure 3:**
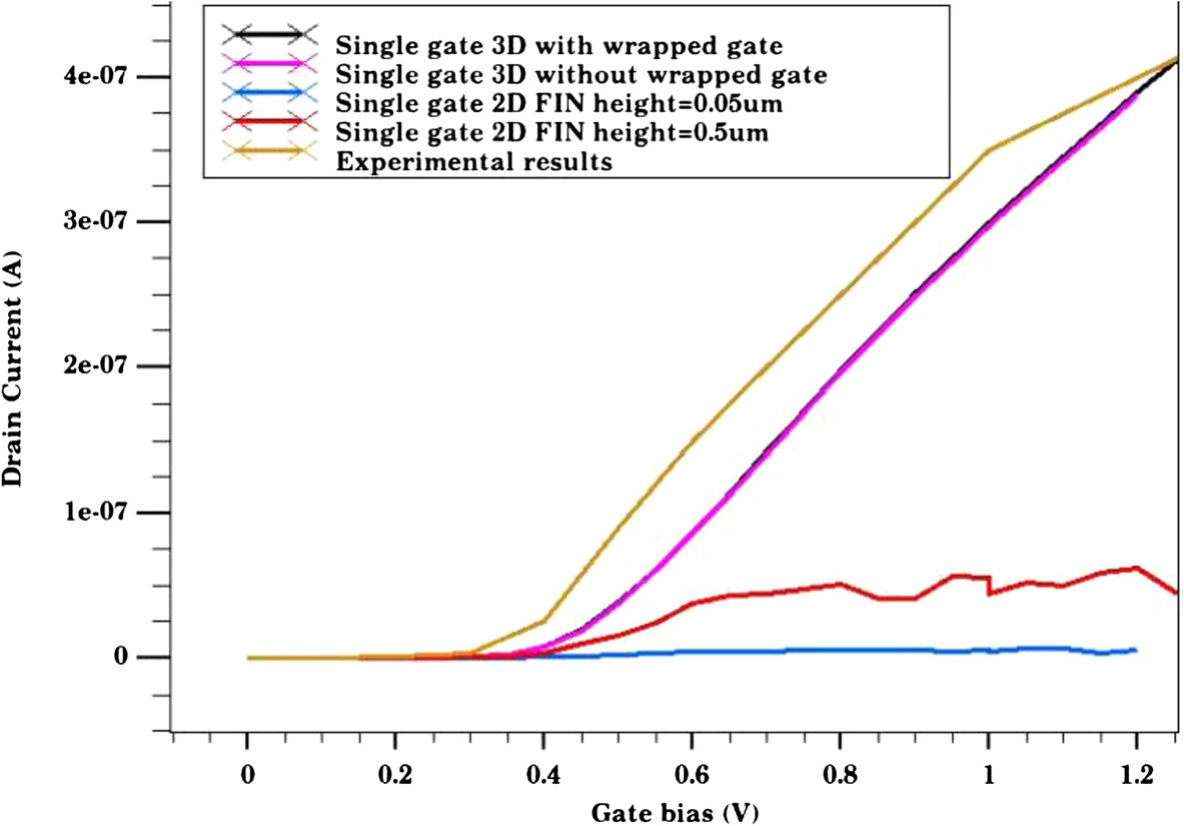
**Comparison of transfer characteristics.** Transfer characteristics for actual device (brown), 3D model with wrapped gate (black), 3D model without the top segment of the gate (purple), 2D model with a 0.5-μm FIN height (red), and 2D model with 0.05-μm FIN height (blue).

In addition to the comparison of electrical field for all the cases described above, we compared their performance characteristics. Figure 3 represents the transfer characteristics for all the three cases. It also shows the transfer characteristic for the 2D model with a fin height 10 times that of the actual fin height. As expected, the current of the 2D model increases 10 times with an increase in FIN height. However, the overall current is far less than that obtained from a 3D model. It can be argued that the 3D model takes into account the 2D quantization as opposed to the 1D quantization speculated in a 2D model. Hence, the overall channel conductance is different in both cases.

### Dual-gate FinFET

It is well established that the device characteristics for most FET devices are largely dependent on the field in the channel. In [10], we presented 2D modeling results for a FinFET with a second gate. The presence of the second gate improves the control of the device, thereby resulting in flatter transconductance. This philosophy was applied to 3D modeling as well to understand the effect of two wrapped gates more accurately. The two gates were of the same length (30 nm) and separated by 30 nm (Figure 1a).

**Figure 4:**
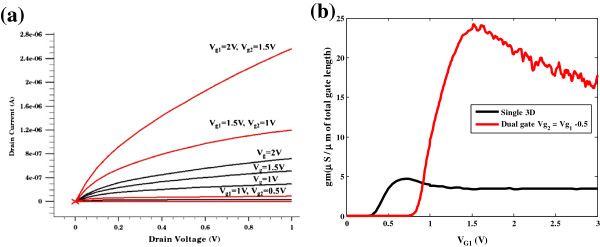
**Single-gate and dual-gate DC characteristics.** (**a**) *I*-*V* characteristics for single-gate (black) and dual-gate FinFET (red). (**b**)Transconductance characteristics for single-gate (black) and dual-gate FinFET (red).

Figure 4a, b represents the current-voltage (*IV*) characteristics and transconductance curves for the dual-gate FinFET. The channel threshold voltage has increased from 0.5 to 1 V with the inclusion of the second gate. Further, the dual-gate FinFET gave a larger transconductance than the single-gate FinFET. This is contrary to our earlier work on the 2D modeling of FinFET [10]. This requires a closer examination of the 3D modeling of dual-gate FinFETs.

## Conclusions

Our study demonstrated that 3D modeling of non-planar devices such as FinFETs is more accurate than 2D models. 3D modeling presents a very detailed electrical field profile. Wrapping the gate around the FinFET channel provides a better control of the device. 3D modeling of transconductance for the single gate showed the value of *g*_m_ to be very close to measured output characteristics of the FinFET. The experimental measurements and 3D modeling produced the same *g*_m_ = 4 μS/μm of the total gate length. However, 3D modeling generated surprisingly high results for transconductance of the dual-gate FinFET.

In our 2D design and modeling of MESFETs, HEMTs and MOSFETs, the device dimensions were in micrometers. The dual gates in these devices produced usually uniform but smaller transconductance [6,10]. One can see on Figure 4 that for dual-gate FinFET, *g*_m_ is not uniform.

We plan to continue our research to find the explanation for the unexpected values and shape of *g*_m_ and RF behavior of the novel FinFET.

## Competing interests

The authors declare that they have no competing interests.

## Authors’ contributions

The concept was initiated by SM. 2D modeling was done by BZ and LD with the aid of JP. Preliminary 3D modeling was done by JP. BZ and LD expanded and completed the 3D modeling. The manuscript was drafted by LD and SM. All authors read and approved the final manuscript.
